# Targeted *β*-therapy with rhenium-aided therapy for cutaneous lesions: a systematic review and meta-analysis

**DOI:** 10.3389/fmed.2025.1707729

**Published:** 2025-11-27

**Authors:** Sajjad Sadeghpour, Ramin Sadeghi, Atena Aghaee, Amin Saber Tanha, Alessio Rizzo, Giorgio Treglia, Saba Sadeghpour

**Affiliations:** 1 Nuclear Medicine Research Center, Mashhad University of Medical Sciences, Mashhad, Iran; 2 Nuclear Medicine Unit, Candiolo Cancer Institute, FPO–IRCCS, Turin, Italy; 3 Faculty of Biomedical Sciences, Università della Svizzera Italiana, Lugano, Switzerland; 4 Faculty of Biology and Medicine, University of Lausanne, Lausanne, Switzerland; 5 Nuclear Medicine, Imaging Institute of Southern Switzerland, Ente Ospedaliero Cantonale, Bellinzona, Switzerland; 6 Madani Hospital, Gonabad University of Medical Sciences, Gonabad, Iran

**Keywords:** skin cancer, rhenium, resin, skin cancer therapy, systematic review, cutaneous lesion, non-melanoma skin cancer

## Abstract

**Background:**

Non-melanoma skin cancer is the most prevalent malignancy. Primary treatments may at times yield suboptimal results. In these cases, alternative therapies or the combination of different modalities may be required to achieve complete removal and prevent the risk of recurrence.

**Methods:**

We examined studies published up to July 2025 using databases such as PubMed and Scopus, focusing on the performance of rhenium-assisted therapies with respect to therapeutic efficacy and potential adverse effects. Studies investigating the therapeutic outcomes of rhenium-assisted therapies for cutaneous lesions were considered. The primary endpoint was the response rate to rhenium-assisted therapies.

**Results:**

This systematic review and meta-analysis included 10 studies involving 433 patients and more than 618 lesions. Of these, seven studies were included in the meta-analysis, in which rhenium-assisted therapy achieved a complete response rate of 88.67%(95% CI: 84.7–91.7%) and an overall response rate of 92.9% (95% CI: 89.1–95.5%). A leave-one-out analysis was performed to assess the study’s impact on heterogeneity, revealing that one study markedly influenced the pooled effect. By excluding this outlier, heterogeneity was substantially reduced, while the overall mean effect remained significant (complete response 94, 95% CI [90, 96%]; overall response 97, 95% CI [92, 99%]).

**Conclusion:**

Rhenium-assisted therapy for skin cancer appears effective and safe in treating lesions unresponsive to standard options. It may represent a valuable tool, providing advantages over conventional approaches. Further studies are warranted to confirm its efficacy and establish standardized protocols.

## 1 Introduction

The World Health Organization estimates that between 2 and 3 million cases of non-melanoma skin cancer occur annually worldwide, although this figure is likely an underestimation (1). The incidence is rising rapidly, primarily due to increased ultraviolet exposure and an aging population (2). The majority of cases are diagnosed at an early stage and are considered low-risk, rendering them suitable for treatment with surgery, pharmacotherapy, or radiotherapy. Radiation therapy has been employed for over a century, initially through the direct application of radium to the skin. Currently, skin radiotherapy is delivered using either external beam radiation or brachytherapy (interventional radiotherapy). It is effective in managing non-melanoma skin cancers, such as basal cell carcinoma (BCC) and squamous cell carcinoma (SCC), and keloids and precancerous cutaneous conditions (3). Non-surgical approaches are particularly suitable when excision may cause significant cosmetic or functional impairment, particularly for older or frail patients with elevated surgical risk or for those who decline operative management (4). Key factors that influence therapeutic choice include the lesion’s location, number, and size, as well as the patient’s immune status, treatment cost, convenience, and personal preferences. In light of the high and increasing global incidence, there is an urgent need to advance non-invasive, patient-centered therapies that provide both convenience and personalized care.

Recently, Re-188 has been introduced in the field of dermato-oncology and is administered as a paste for cutaneous application. Unlike the sealed isotopes conventionally employed in brachytherapy, Re-188 is utilized as an unsealed isotope. Other *β*-emitters, including ^90^Y (5), ^32^P (6), and ^166^Ho (7), have also been proposed for the treatment of skin cancer and are at various stages of development.

This systematic review and meta-analysis aimed to evaluate the existing evidence from published trials to determine the response rate of rhenium-based therapy for cutaneous malignant lesions. Secondary endpoints included the assessment of adverse effects and their clinical relevance. By synthesizing current data, this review sought to elucidate the therapeutic role of Re-188 in skin cancer and to highlight areas requiring further investigation.

## 2 Methods

### 2.1 Study design and data sources

The present systematic review and meta-analsysis was conducted following the PRISMA protocol (8) and was structured around the following patient, intervention, comparator, outcome (PICO) framework: Patient: Individuals diagnosed with skin cancerous lesions; Intervention: Treatment with Re-188 resins or rhenium-labeled antibodies; Comparator: Any alternative therapeutic option, when studies included a comparator arm; and Outcome: Response rate and the frequency of adverse events.

Comprehensive searches of PubMed and Scopus were conducted using the following keywords: A: “Rhenium” OR “186Re” OR “Re-186” OR “Rhenium-186” OR “188Re” OR “Re-188” OR “Rhenium-188”; B: “Skin Neoplasms” OR “skin cancer” OR “cutaneous neoplasm*” OR “cutaneous carcinoma” OR “non-melanoma skin cancer” OR “basal cell carcinoma” OR “squamous cell carcinoma” OR melanoma; C: “Therapeutics” OR therapy OR treatment OR “therapeutic” OR “efficacy” OR “clinical trial.” No restrictions were applied regarding the publication date. Only studies published in English that focus on the treatment of skin cancer lesions with rhenium were included. To ensure thorough coverage, the reference lists and citing articles of the retrieved studies were examined for additional relevant studies. The most recent search update was performed on 30 July 2025.

The eligibility criteria mandated that human studies included adequate data on patient demographics and cancer subtypes. Reviews, editorials, case reports, and conference abstracts were excluded from the systematic review to ensure methodological rigor. When duplicates were identified, only the latest or most comprehensive publication was included, and duplicate studies were omitted whenever possible.

### 2.2 Data extraction

Data extraction was performed using a standardized form to collect information on patient numbers, lesion sites and counts, lesion subtypes, response criteria and assessment methods, prior treatments, response rates, tumor staging and classification, the type of rhenium compound administered, activities, margins of clinically normal skin included, number of sessions, follow-up duration, and adverse effects.

Risk of bias (RoB) was assessed using the ROBINS-I tool for non-randomized studies of interventions. This tool evaluates seven domains: bias due to confounding, bias in participant selection, bias in the classification of interventions, bias due to deviations from intended interventions, bias due to missing data, bias in outcome measurement, and bias in the selection of the reported results, leading to an overall judgment of risk. The preferred reference standard was histological confirmation, which is considered the gold standard for diagnosis.

### 2.3 Meta-analysis

Meta-analysis was performed using the Meta-MUMS software (9) with a random effects model (DerSimonian and Laird method) (10). This approach accounts for variability across studies, making it suitable for combining data from diverse research studies. Heterogeneity was assessed using the Cochran’s Q test (with *p* < 0.05 indicating significance) and quantified using the I^2^ index, which represents the percentage of variability due to actual differences rather than sampling error.

Publication bias was evaluated using funnel plots, Egger’s regression intercept, and the Duval–Tweedie trim and fill method (11). In funnel plots, the standard errors of the studies were plotted on the x-axis and their effect sizes on the y-axis. If these plots showed asymmetry, it suggested potential publication bias, which was further quantified using Egger’s regression intercept (*p* < 0.05 indicated significant bias). Finally, the Duval–Tweedie trim and fill method addressed this bias by iteratively removing smaller studies to achieve symmetry, yielding an adjusted pooled effect size that reflected the potential influence of publication bias. The key treatment index included the response rate.

## 3 Results

### 3.1 Literature search and study selection

To provide a comprehensive overview of the literature on Re-188 application for cutaneous lesions, both Re-188 resin and rhenium-labeled antibodies were included and evaluated in this study. The broader term rhenium-aided therapy was adopted for clarity and consistency. However, regarding the meta-analysis, only one study discussed a rhenium-labeled antibody and its application (12); therefore, it was excluded. This is particularly relevant considering that the main use of rhenium in the treatment of cutaneous tumors generally involves the application of resins rather than the administration of radiopharmaceuticals.

**Figure 1:**
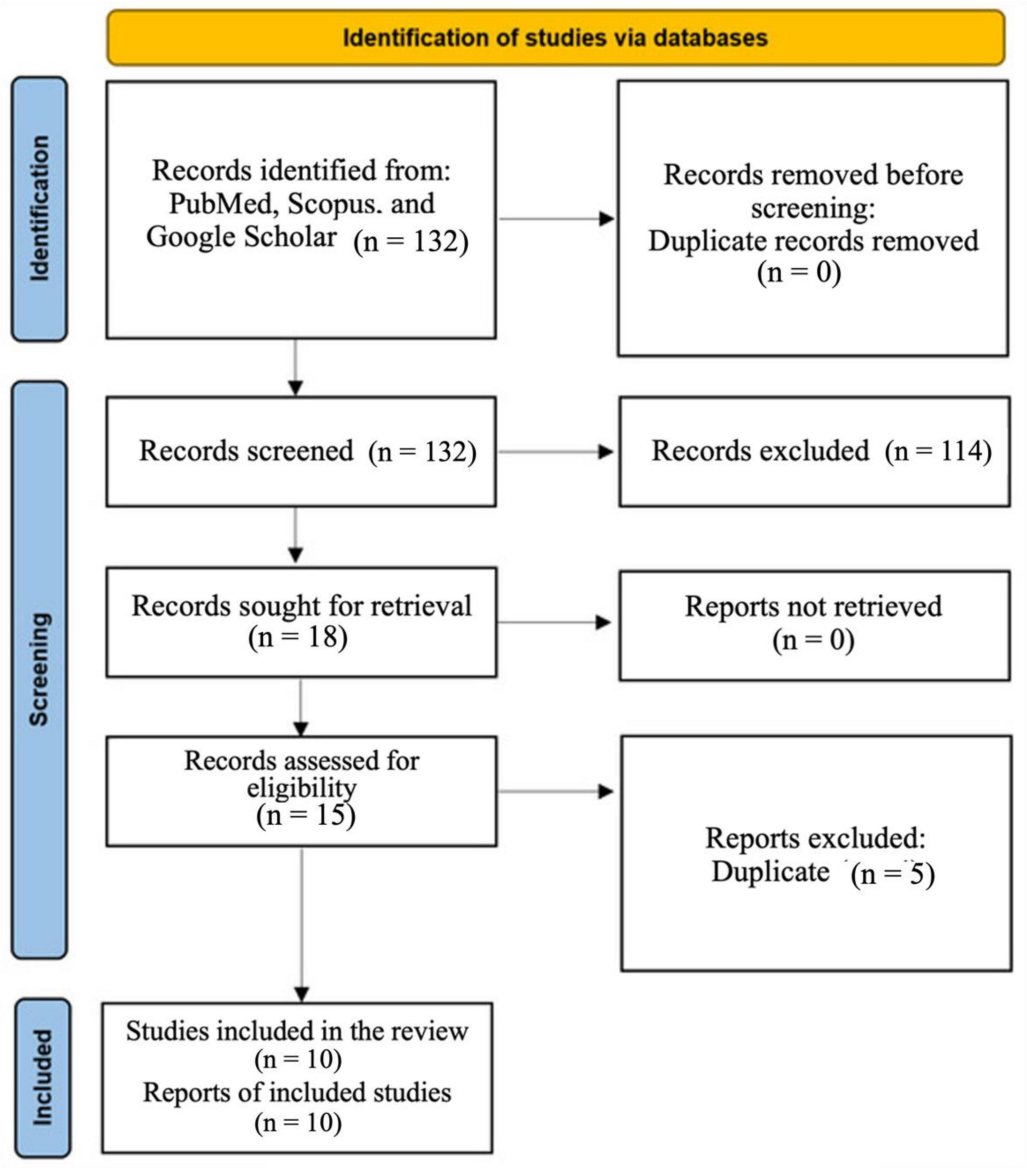
PRISMA flow diagram.

Of the 132 primary articles found, 15 were selected for inclusion. Similar studies conducted at similar centers with comparable recruitment periods were evaluated for potential overlap. Of the 15 studies, five were excluded due to overlapping data or patient exclusion (13–17). Of the three studies conducted by colleagues affiliated with the Azienda Ospedaliero-Universitaria di Bologna, Sant’Orsola-Malpighi Hospital, Italy (15, 18, 19), all were investigated for overlap across different cancer types and recruitment periods. One of them was excluded (15), while the other two showed no overlap and were retained (18, 19). Furthermore, three other studies (13, 20, 21), registered under the EPIC-Skin trial (trial code NCT05135052), were investigated; one (13) was excluded due to overlap. A total of two studies conducted at S. Eugenio Hospital, Rome (16, 22), were also examined, and potential overlap was ruled out (Figure 1).

### 3.2 Qualitative synthesis

#### 3.2.1 General characteristics

Following the screening of studies from the same centers with overlapping recruitment periods, this review synthesized evidence from 10 investigations. In total, five were conducted in Italy, and one each in South Africa, India, the USA, and Germany, in addition to one multicenter study. The included studies addressed a spectrum of cutaneous conditions, including squamous cell carcinoma (SCC), basal cell carcinoma (BCC), metastatic melanoma, extramammary Paget’s disease, Bowen’s disease, and keloids. Sample sizes ranged from 5 to 140 participants, and lesion counts per study ranged from 5 to 185. In total, 477 patients and more than 618 lesions were evaluated (Table 1).

**Table 1:** Characteristics of the included articles.

Study	Center	Study design	Patients	Lesions	Response definition and evaluation	Sub-populations	Age	Gender distribution	lesions	Tumor staging	Location	Prior Tx	Used Re compound	Absorbed Dose	Margin (mm)	Cycles	F/U	Funding source
Cipriani et al. (1)	Italy, Rome	Retro	52	55	NS (clinical examination)	BCC (32), SCC (19), Bowen (1), EMPD (1)	72	34 M, 18 F	55	NS	72.3% H&N, trunk (1.8%), limbs (10.9%), genitalia (14.5%)	28/52 (surgery, etc.)	Rhenium-188 resin (acrylic matrix)	50Gy (target), 20–100 MBq/cm2	5	1	296 D	OncoBeta (Bavarian research program grant)
Cardaci et al. (2)	7 centers	Pros phase 4	140	185	CR: disappearance, PR: minimum 30% reduction in largest diameter, PD: minimum 20% increase in largest diameter, SD: between PR and PD	BCC (148), SCC (37)	70		185	Stage I/II, ≤3 mm thickness, ≤8 cm^2^ area	H&N (67.3%), upper limb (9.1%), lower limb (11.8%), torso (11.8%)	without any prior Tx (surgery, radiation, laser therapy)	Rhenium-188 resin (adhesive foil)	50Gy (target)	5	1	12 mo	OncoBeta Therapeutics Australia
Tietze et al. (3)	Germany	Pros	22	40	NS (clinical examination)	Bowen (12), BCC (23), SCC (5)	83	11 M, 11 F	40	≤3 mm thickness	Face (27.5%), head (25%), trunk (22.5%), lower extremity (25%)	Imiquimod (4), surgery (3), diclofenac gel + curettage	188Re resin (OncoBeta®)	50Gy (target), 111.4 MBq	5	1	12 mo	German Research Foundation, BMBF, BMWiE, Oncobeta® GmbH
Zagni et al. (4)	Bologna, Italy	Retro	75	94	NS	BCC (57), SCC (37)	82	47 M, 28F	94	≤3 mm thickness	Scalp (19%), face (29%), nose (22%), ears (10%), body (20%)	NS	188Re resin (Oncobeta®)	23.5Gy(target), 48.3Gy	3			Italian Ministry of Health (RC-2023-2778829)
Klein et al. (5)	USA	Trial phase Ia/Ib	20	NS	RECIST v1.0	metastatic melanoma	64	15 M, 5F	NS	Stage IIIC/IV	Various metastatic sites	Chemotherapy (14), immunotherapy (15), RT (7), surgery (20)	^188^Re-labeled antibody (6D2)	10 mCi (adminstered)		1	OS: 13 mo	Pain Therapeutics, Inc.
Chessa et al. (6)	Italy.	Retro	64	82	CR: without any suspected area of relapse in videodermoscopy or dermoscopy -guided negative biopsy. Non-responder: relapses by positive histological exam.	BCC (60 nodular; 9 sclerodermiform; 13 superficial)	82	40 M, 24 F	82	≤3 mm thickness	Face/scalp 50%; trunk/extremities 33.3%; low-risk areas 16.7%	Surgery (3), PDT/cryotherapy (6), cryotherapy/laser/PDT (10), imiquimod (1)	Non-sealed 188 Re resin (Rhenium-SCT®)	NS	3	1	time to relapse: 24 mo	NS
Mokoala et al. (7)	South Africa	Retro	29	58	No response: < 20% reduction in height and/or diameter. PR: reduction of between 20 and 70% of the height and/or diameter. CR: > 70% reduction in height and/or diameter. PD: An increase in height or diameter.	recurrent Keloid	40	14 M, 15 F	58		H&N: 62.0%; trunk: 24.1%; limbs: 15.5%	Surgery, intralesional steroids, RT	dirhenium-heptasulfide in jelly matrix	30 Gy (target), 256.7 MBq	NS	1–2	37 mo	Open access funding by University of Pretoria
Carrozzo e al. (8)	Italy,	NS	14	14	NS (clinical examination with dermoscopic and histological evaluation)	penile SCC	66	all men	14	Tis (9), Ta (3), T1a (3)	Glans (majority), penile shaft (1)	Topical therapy (steroids, antibiotics, hormones), imiquimod, 5-FU	Synthetic inert resin-matrix with 188-Re nanocolloid	65Gy	2–4	1–7	51 mo	NS
Bhusari et al. (9)	India	NS	12	85	NS (gross examination)	keloids	39	9 M, 3F	85		Multiple locations (surgical keloids, scratch keloids, acne-related keloids)	Steroid injections, silicon bandage, other therapies (unspecified)	Re-188 colloid patch	100Gy/mCi, 1481Gy	NS	1–4	1–3 y	PGIMER, Chandigarh
Carrozzo et al. (10)	Italy	NS	5	5	NS (clinical examination and histological evaluation)	EMPD	69	1 M, 4F	5	4 primary, 1 secondary	Glans, perineal/vulvar., vulvar., perivulvar regions	Topical therapies (unspecified), steroid creams	Re-188 in synthetic matrix	50Gy (target)	2–4	1–2	34 mo	NS

Rtro: Retrospective, Pros: Prospective, EMPD: Extramammary Paget Disease, M: Male, F: Female, PDT: Photodynamic Therapy, H&N: Head & Neck, NS: Not Specified, RT: Radiation Therapy.

#### 3.2.2 Risk of bias

All included studies demonstrated a low to moderate RoB across most domains and overall. The confounding domain was generally rated as low to moderate risk; however, several studies raised serious or critical concerns. Therefore, although the majority of investigations maintained a low to moderate RoB profile, a subset exhibited limitations related to confounding, necessitating cautious interpretation of their findings (Table 2; Supplementary Figure 1).

**Table 2:** Risk of bias assessment of the included studies using the ROBINS-I checklist.

Author	Confounding	Selection	Classification of interventions	Deviations from interventions	Missing data	Outcome measurement	Reporting bias	Overall
Cipriani et al. (1)	Moderate. Retrospective, without adjustment for prior treatments or comorbidities.	Moderate. 52 patients selected over 9 years; potential selection bias.	Low. Clear treatment protocol.	Low. Single-session therapy.	Moderate. 14/52 lost to follow-up; reasons unclear	Low. Objective clinical remission assessment	Low. Consistent reporting of outcomes	Moderate
Cardaci et al. (2)	Low. Prospective; predefined eligibility	Low. Consecutive enrollment; multicenter	Low. Standardized protocol.	Low. Single-session therapy.	Moderate. 23.9% lost to follow-up at 12 months	Low. Validated scales: CTCAE v5.0	Low. Predefined primary/secondary endpoints.	Moderate
Tietze et al. (3)	Moderate. Small sample, without control group	Moderate. Convenience sample; some lesions excluded	Low. Clear dosimetry protocol.	Low. Single session.	Low. 1/22 lost due to death; minimal attrition.	Low. Biopsy-confirmed responses.	Low. Detailed cosmetic/clinical outcomes.	Moderate
Zagni et al. (4)	Low. Comparing Monte Carlo-based tool with VARSKIN and experimental validation, controlling for target dimensions and activity.	Moderate. Retrospective cohort of 76 patients; selection may not fully represent all NMSC cases.	Low. Clear description of Re-188 resin application and dosimetry methods.	Low. No deviations reported; standardized protocol followed.	Low. Complete data for dosimetric comparisons.	Low. Objective outcomes (dose distribution, Gafchromic film measurements).	Low. All outcomes (dose agreement, validation) reported.	Low
Klein et al. (5)	Moderate. Without adjustment for prior therapies or metastatic burden in survival analysis.	Moderate. Small sample (20 patients); eligibility criteria may limit generalizability.	Low. Clear dosing and administration of ^188^Re-6D2.	Low. No deviations reported.	Moderate. Incomplete follow-up data for some patients (e.g., HAMA response).	Low. RECIST criteria and SPECT/CT for tumor response.	Moderate. *Post hoc* survival analysis not pre-specified.	Moderate
Chessa et al. (6)	Moderate. Without adjustment for lesion heterogeneity in relapse analysis.	Moderate. Possible selection bias (e.g., only difficult-to-treat BCCs).	Low. Standardized ^188^Re brachytherapy protocol.	Low. Consistent treatment application.	Low. Complete follow-up for primary outcomes.	Moderate. Dermoscopic assessment of relapse subjective (false positives noted).	Low. All outcomes (efficacy, relapse) reported.	Moderate
Mokoala et al. (7)	Moderate. Lack of adjustment for potential confounders (e.g., prior treatments, lesion size).	Low. Consecutive patient recruitment, Clear predefined eligibility criteria.	Low. Standardized treatment application using the Oncobeta device.	Low. No deviations reported; all patients received the planned therapy.	Moderate. Follow-up was irregular for some patients (telephonic follow-up), and quality-of-life data were incomplete.	Low. Objective measures (lesion size reduction) were used, but subjective symptoms were self-reported.	Low. All outcomes (response rates, recurrence) were reported.	Moderate
Carrozzo et al. (8)	Critical. No adjustment for confounders (e.g., HPV status, prior treatments). Heterogeneity in tumor stages (Tis–T1a) and sessions (1–7).	Moderate. Small sample (n = 15) with unclear recruitment methods.	Low. Clear protocol for rhenium brachytherapy application.	Low. No deviations reported.	Moderate. One patient lost to follow-up; others had variable follow-up durations (24–84 months).	Low. Histological confirmation of remission was used.	Low. Outcomes (remission, non-response) were reported.	Critical
Bhusari et al. (9)	Moderate. No control group; confounders (e.g., prior treatments) were present but not adjusted for.	Low. Consecutive recruitment from a single center, no exclusion criteria mentioned.	Low. Clear description of Re-188 patch application (dose, duration).	Moderate. No protocol deviations reported, but adherence not explicitly monitored.	Low. Complete follow-up data for all patients at specified intervals.	Moderate. Subjective assessment of keloid size/symptoms; no blinding.	Low. All outcomes (symptom relief, size reduction) reported.	Moderate
Carrozzo et al. (10)	Serious. No control group; confounders (e.g., prior treatments, disease stage) unaddressed.	Moderate. Small sample (*n* = 5); unclear selection criteria.	Low. Detailed intervention (dose, technique) described.	Moderate. No protocol deviations reported, but lack of adherence monitoring.	Low. Complete follow-up data for all patients.	Moderate. Clinical/histological outcomes assessed without blinding.	Low. Consistent reporting of outcomes (healing, relapse).	Serious

#### 3.2.3 Response criteria and evaluation

Of the 10 studies assessed, only four employed predefined response criteria and explicitly reported them, using precise metrics such as changes in lesion size and/or diameter. A total of two studies applied a combination of clinical examination and histological evaluation for patient follow-up. The remaining four studies did not specify the criteria adopted and reported outcomes based on clinical examination in a general, non-standardized manner (Table 1). A key factor influencing response measurement is how the response is defined, which varies from solely clinical examination (20, 22, 23) to more standardized radiological criteria, such as RECIST v1.0 (24) or confirmation through dermoscopy or histology (19, 22). Studies using stricter histological or dermoscopic endpoints tended to report lower complete response rates (CRRs), likely reflecting more sensitive detection of subclinical disease persistence or recurrence. Moreover, in many protocols, the margin of clinically normal skin treated with rhenium resin was set at 5 mm; however, this parameter was not consistently reported, contributing to variability in local control outcomes.

#### 3.2.4 Adverse events

Absorbed dose, number of cycles, and follow-up duration also significantly affected therapeutic outcomes. Most cutaneous carcinoma protocols targeted a 50 Gy dose in a single session (12, 20, 21, 23, 24), but there were variations: Zagni et al. reported lower doses (23–48 Gy), which could impact long-term clearance, while protocols for keloid and penile SCC used higher cumulative doses or repeated cycles (22, 25). Follow-up periods varied, ranging from less than 1 year in some prospective studies (12 months) to over 4 years in cases of penile SCC and EMPD (22, 26), with longer follow-up revealing a higher number of recurrences. In addition, prior treatments, such as surgery, imiquimod, photodynamic therapy, or radiotherapy, were common in retrospective cohorts. They could have influenced responses, especially since treatment-resistant or recurrent lesions were often included (Supplementary Table 1).

### 3.3 Meta-analysis

#### 3.3.1 Complete response rate (CRR)

**Figure 2:**
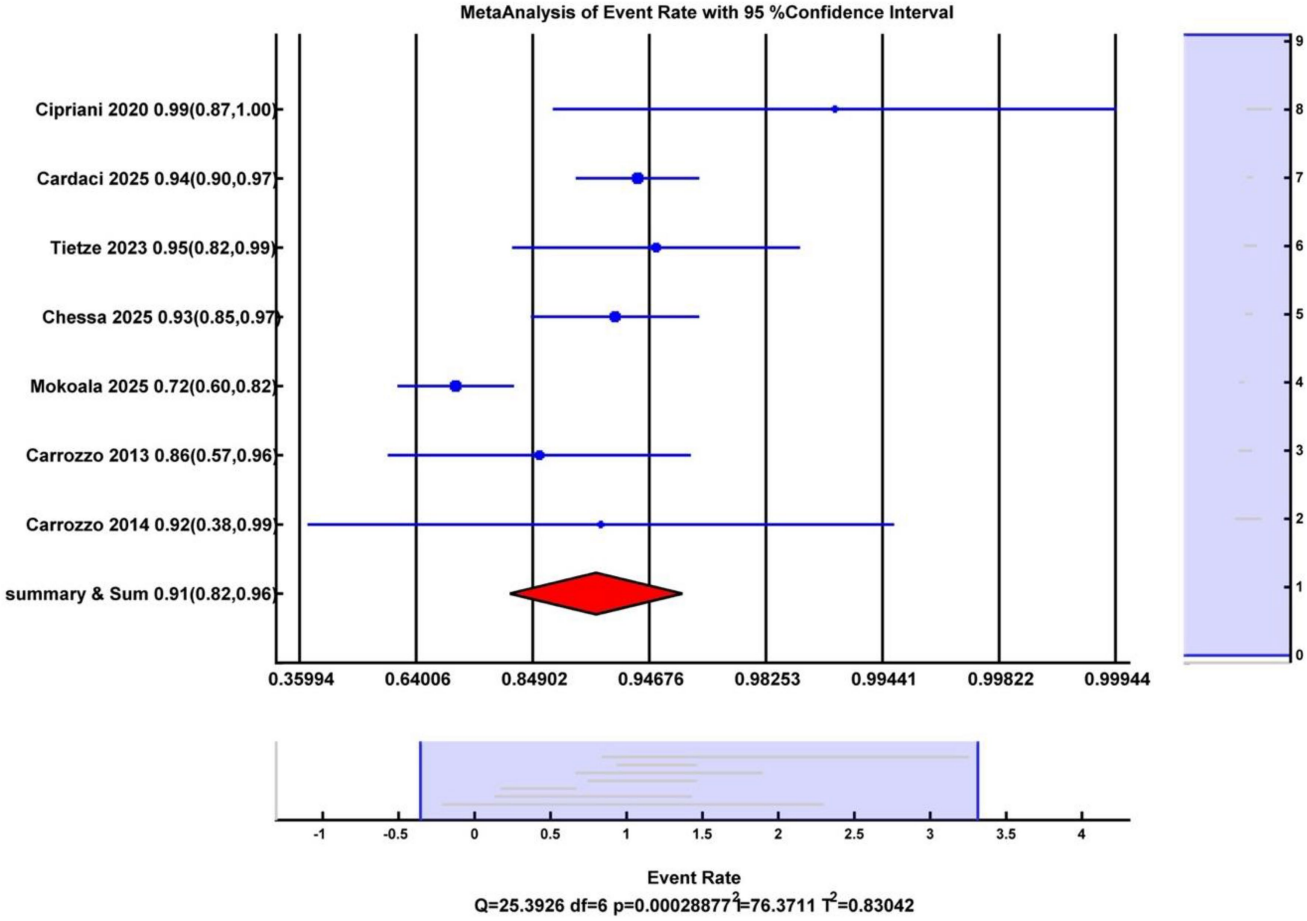
Forest plot illustrating the complete response rate across the included studies.

A total of eight studies were included in the meta-analysis to calculate the CRR of rhenium for skin cancerous lesions. The pooled complete response rate for rhenium-aided skin cancer therapy was 88.67% (95% CI: 84.70–91.71%) (Figure 2). Heterogeneity analysis indicated a Cochran’s Q value of 25.39 (*p* < 0.001) and an I^2^ index of 76.37%.

**Figure 3:**
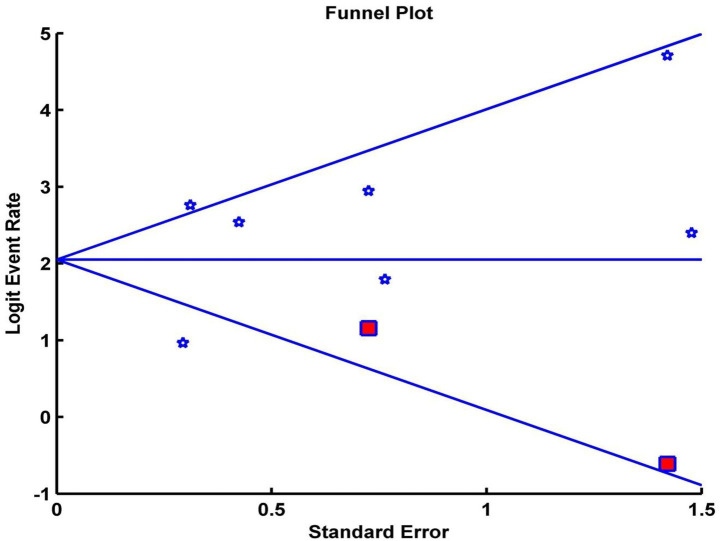
Funnel plot of the pooled complete response rate. White asterisks represent the included studies. Red squares represent the studies trimmed to correct for asymmetry. The black diamond represents the adjusted pooled effect size accounting for potential publication bias, calculated using the Duval–Tweedie trim and fill method.

Analysis of publication bias using Egger’s test and the trim and fill adjustment did not significantly change the CRR, indicating that small-study effects were unlikely to influence the outcome. Egger’s regression intercept was calculated at 1.56 (*p* = 0.36). After applying Duval–Tweedie’s trim and fill method and removing two studies, the funnel plot became symmetrical, yielding an adjusted pooled CRR of 88.60 (95% CI: 78.05–94.43%), representing a 0.07% reduction from the original pooled CRR (Figure 3).

Sensitivity analyses were conducted to examine the influence of individual studies on heterogeneity. A leave-one-out analysis indicated that a single study substantially altered the overall effect size. The exclusion of this outlier study reduced heterogeneity from high (*I*^2^ = 76.37%, *Q* = 25.39, *p* < 0.001) to low (*I*^2^ = 0.0%, *Q* = 3.74, *p* = 0.58), while the overall mean effect remained significant (CRR = 94, 95% CI [90, 96%]). Given the small number of included studies, tests for publication bias, including funnel plot inspection and Egger’s test, have low statistical power and should be interpreted as exploratory. These results suggest that the findings were preliminary and that heterogeneity was partly attributable to a single outlying study.

#### 3.3.2 Overall response rate (ORR)

**Figure 4:**
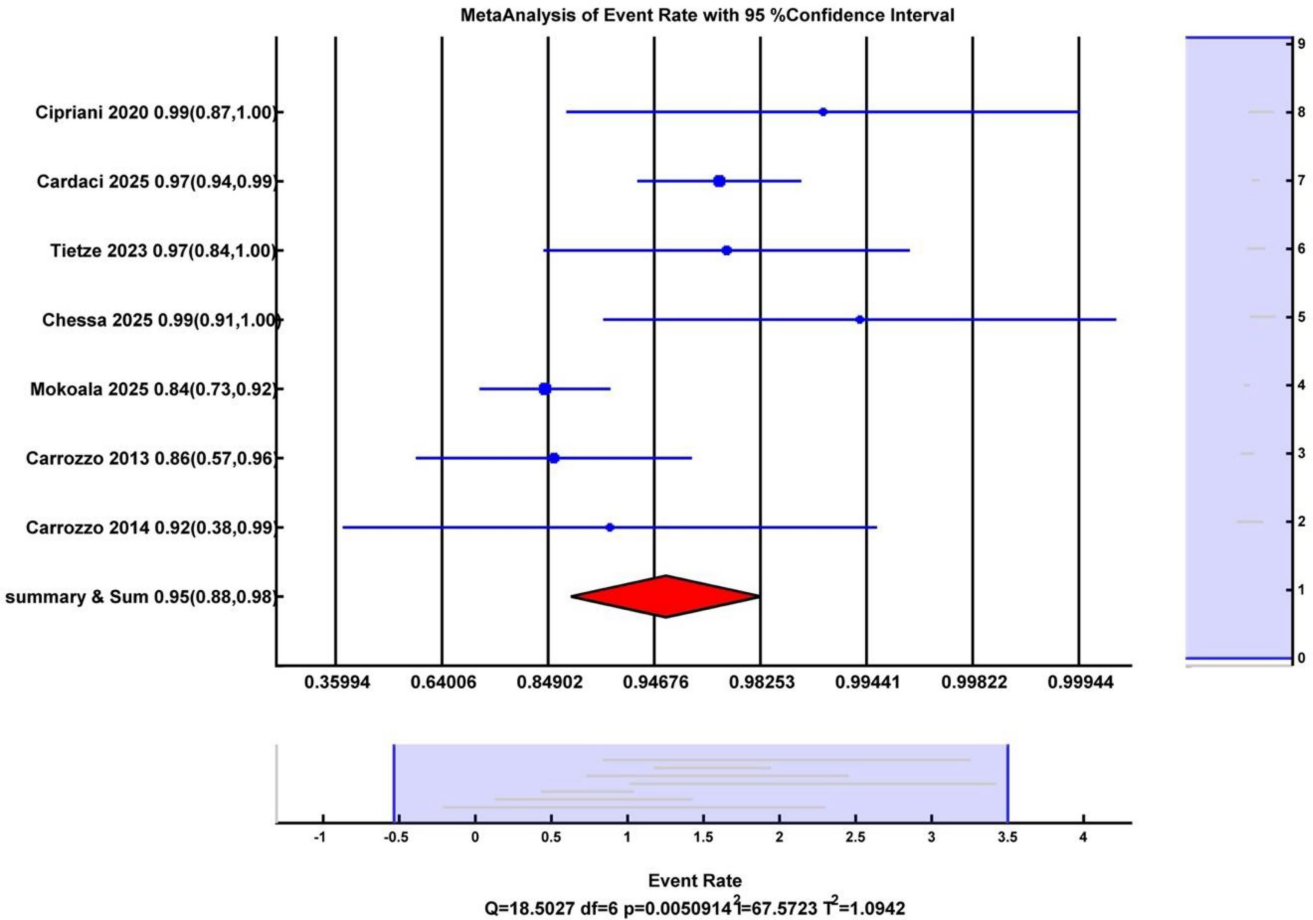
Forest plot illustrating the pooled overall response rate across the included studies.

The overall response rate (ORR) was defined as the complete response rate plus the partial response rate. A total of eight studies were included in the meta-analysis to calculate the ORR of rhenium for skin cancerous lesions. The pooled overall response rate of rhenium-aided skin cancer therapy was 92.99% (95% CI: 89.13–95.55%) (Figure 4). Heterogeneity analysis indicated a Cochran’s Q value of 18.50 (*p* = 0.005) and an I^2^ index of 67.57%.

**Figure 5:**
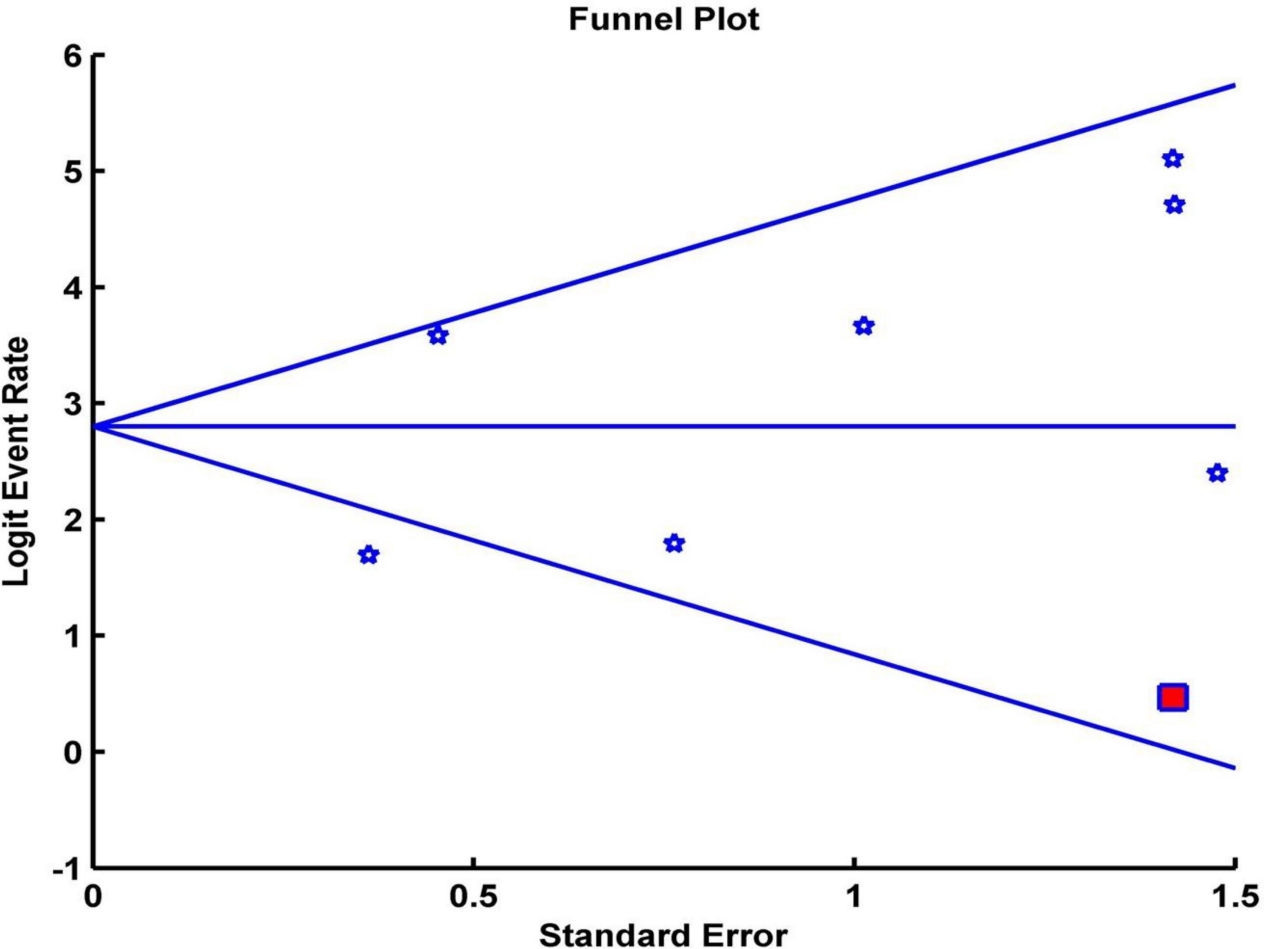
Funnel plot of the pooled SUV. White asterisks represent the included studies. Red squares represent the studies trimmed to correct for asymmetry. The black diamond represents the adjusted pooled effect size accounting for potential publication bias, calculated using the Duval–Tweedie trim and fill method.

Analysis of publication bias using Egger’s test and adjustment with the trim and fill method did not significantly change the ORR, indicating that small-study effects were unlikely to influence the outcome. Egger’s regression intercept was calculated at 1.67 (*p* = 0.237). After applying Duval–Tweedie’s trim and fill method and removing one study, the funnel plot became symmetrical, yielding an adjusted pooled ORR of 94.27 (95% CI: 85.81–97.81%), representing a 1.28% reduction from the original pooled CRR (Figure 5).

Sensitivity analyses were conducted to examine the influence of individual studies on heterogeneity. A leave-one-out analysis indicated that a single study substantially altered the overall effect size. The exclusion of this outlier study reduced heterogeneity from high (*I^2^* = 67.57%, *Q* = 18.50, *p* = 0.005) to moderate (*I^2^* = 32.20%, *Q* = 7.38, *p* = 0.19), while the overall mean effect remained significant (ORR = 97, 95% CI [92, 99%]). These results suggest that the findings were preliminary and that heterogeneity was partly attributable to a single outlying study.

## 4 Discussion

When interpreting these results, it is essential to recognize that the included studies employed heterogeneous criteria for lesion assessment, ranging from gross clinical inspection to histological confirmation, particularly when evaluating different lesion types such as BCC and keloids. This systematic review and meta-analysis highlight the promising potential of rhenium-based therapy as a targeted approach for skin cancer lesions, especially non-melanoma types such as BCC and SCC. The combined CRR of 88.67% (95% CI: 84.70–91.71%) and ORR of 92.99% (95% CI: 89.13–95.55%) indicate strong effectiveness in inducing tumor remission across the included research. These results support rhenium-assisted skin cancer treatment as an effective solution for various lesions, including BCC, SCC, and keloids, particularly for cases that are unresectable or unresponsive to other therapies. Nonetheless, the significant heterogeneity (*I*^2^ = 76.37% for CRR and 67.57% for ORR) observed points to outcome variability, likely due to differences in lesion features, treatment protocols, and patient groups. Sensitivity analyses showed that a single outlier study largely drove this heterogeneity; removing it significantly reduced *I*^2^ to 0.0% for CRR and 32.20% for ORR while still maintaining high effect sizes (94 and 97%, respectively). Tests for publication bias, including Egger’s test (*p* = 0.36 for CRR; *p* = 0.237 for ORR) and trim and fill adjustments, suggested minimal influence from small studies, with adjusted estimates changing only slightly (88.60% for CRR; 94.27% for ORR). These findings confirm the promising nature of the pooled results; however, since they are derived from preliminary, uncontrolled studies, they should be interpreted with caution.

Rhenium-aided therapy works through the targeted delivery of *β*-radiation, making it effective for superficial skin lesions due to its limited tissue penetration (20). As a high-energy β-emitter, Re-188 delivers electrons with a limited penetration range of approximately 3 mm, inducing DNA damage and subsequent cell death predominantly within the tumor microenvironment, while largely sparing deeper healthy tissues (27). This localized cytotoxicity is achieved by applying Re-188 in a resin or paste form, known as Rhenium Skin Cancer Therapy (Rhenium-SCT), which adheres to the lesion surface and allows dose customization based on lesion size, thickness, and location (13). In addition to direct radiation-induced cytotoxicity, evidence indicates an immunomodulatory effect, whereby therapy may activate local immune responses through the release of tumor-associated antigens from dying cells, thereby contributing to sustained tumor control. In a broader radiobiological context, this phenomenon has been described as the abscopal effect, wherein local irradiation induces systemic anti-tumor responses at sites distant from the treated lesion. For cutaneous disease, this mechanism is particularly relevant in multifocal or recurrent conditions, such as keloids or non-melanoma skin cancers, where surgery may impose considerable cosmetic or functional limitations (28). However, effectiveness varies with lesion depth; thicker lesions may require adjusted administered activities to ensure proper dose absorption. In most studies, a target dose of 50 Gy was estimated for lesion depths of 300–600 μm, as *β*-particles lose energy quickly, limiting their effectiveness in deeper tumors.

Given the promising response rates and minimal adverse effects, several variables warrant consideration. These include the type of lesion (e.g., BCC, SCC, or keloid); its location, with particular distinction between cosmetically and functionally sensitive areas, such as the head, neck, and face versus the trunk and limbs; lesion depth, noting that most studies investigated lesions measuring less than 3 mm; the margin of apparently normal skin treated with rhenium-aided therapy; previous treatments, distinguishing between therapy-naïve lesions and those refractory to prior interventions; and the treatment protocol itself, whether administered as single-session high-dose brachytherapy or as a fractionated regimen over multiple sessions.

Regarding lesion type, as shown by several studies (25, 28), keloids, particularly recurrent keloids, have a lower response rate compared to BCC and SCC. This is reflected in the pooled CRR and ORR for all lesions (88.67 and 92.99%), compared to non-keloid lesions (94 and 97%). BCC subtypes can also exhibit different responses; in a study (19), 82 BCC lesions—60 nodular, nine sclerodermiform, and 13 superficial—were evaluated. The results of this study showed a 93% CRR (76/82) and six relapses among sclerodermiform lesions, indicating that the sclerodermiform subtype had a sevenfold higher risk of relapse compared to other subtypes.

The location of the lesions can be important, possibly because the thickness of the corneum layer of the epidermis varies across the body; it is thicker on the torso and limbs and thinner on the face and head. However, this cannot be easily explained because most trials treated heterogeneous lesions from different locations. A total of two studies evaluating exclusively genital and perineal lesions reported complete response rates in most cases after a single session and in others by the second session or with salvage therapy (22, 26), suggesting the greater potential of genital/perineal lesions for this treatment. On the other hand, when evaluating the efficacy of high-dose brachytherapy in non-surgical candidates with BCC, no statistically significant difference in response to therapy was found across anatomical areas (19). This may be explained by the small sample sizes for each area in this study.

Due to the biophysical properties of Re-188, its *β*-particles do not effectively penetrate tissues deeper than 3 mm (27). Studies evaluating lesions less than 3 mm thick suggest that those with deeper involvement or metastasis may not respond to this therapy. Re-188’s beta range (~3 mm) suits superficial targets, but success declines with thicker lesions. Emerging Monte Carlo-based tools, which may reduce both under-treatment (deep margins) and over-treatment (adjacent normal skin), are noteworthy for their potential use in routine clinical practice to calculate the Re-188 resin activity and treatment parameters necessary for obtaining the prescribed minimal target dose (7, 18).

According to the concept of field cancerization, the genetic and epigenetic changes that occur in tumor suppressor genes and proto-oncogenes in cancerous cells can also appear, to some degree, in peripheral and adjacent normal-appearing tissue without being clinically visible (29, 30). Therefore, it seems necessary to cover a normal-appearing margin of the lesion, which ranges from 2 to 4 mm in some studies (18, 19, 22, 26) to 5 mm in others (20, 21, 23). For lesions at higher stages (T in TNM staging), possibly covering a wider normal margin can help reduce the risk of recurrence. However, current evidence does not show any statistically significant differences across various tumor sizes (19).

Theoretically, lesions that respond poorly to prior treatments (e.g., steroids, imiquimod) might do so because radiation penetration is hindered by scar tissue, altered vascularity, or the presence of more aggressive and invasive tumor cells. However, this hypothesis has been examined in only a limited number of studies, which have not demonstrated significant differences in outcomes between previously treated and treatment-naïve lesions (19). Another study assessed exclusively lesions that had not undergone prior therapy, despite being eligible candidates. The findings demonstrated an overall response rate (ORR) of 94.05%, indicating that Re-188 resin therapy yields promising outcomes, even when administered before surgery or as an alternative to other treatments (20).

Although no study directly compared single-session high-dose brachytherapy with fractionated multi-session therapy, available evidence from studies using different protocols suggests that both approaches are comparable in efficacy and tolerability. Several investigations reported applying rhenium-aided skin cancer therapy for as long as it remained effective without inducing significant adverse effects (22, 25, 26). In one study, the sessions were extended up to seven for SCC (22). Another study applied up to four sessions and an accumulated dose of 3,774 Gy for keloids (25). A study used two sessions with a target dose of 50 Gy each (at a depth of 300 μm) for genital/perivulvar EMPD (26).

When evaluating adverse events, factors such as the absorbed dose, number of fractions, treatment duration, inter-session interval, and timing of follow-up should be considered. Regarding safety, adverse events associated with rhenium-aided skin cancer therapy are generally mild and self-limiting, reflecting its topical and non-invasive nature. The most frequently reported side effects include grade 1–2 radiation dermatitis (up to 88%), hypopigmentation (up to 60.4%), erythema, pruritus, and pain, all of which typically resolve within weeks to months (14, 31). Severe adverse events, such as grade 3 ulcers, are infrequent, as illustrated by a single case reported by Tietze et al. (21), and are typically associated with larger or thicker lesions, underscoring the importance of careful patient selection and accurate dosimetry. No dose-limiting toxicities have been reported with antibody-labeled formulations.

These findings suggest that rhenium-aided skin cancer therapy is a promising alternative to surgery or external beam radiotherapy, especially for older patients or those with comorbidities, where minimizing procedural risks is vital (20). Nonetheless, the variation in reported adverse events, from burning sensations and crusting to arborizing vessels, highlights the need for standardized monitoring in future research.

## 5 Limitations and future directions

Regarding the practicality of this method, the ^188^W/^188^Re generator produces carrier-free ^188^Re (as sodium perrhenate) through the decay of tungsten-188 (half-life of approximately 69 days). It requires elution with saline, followed by chemical processing to create a ^188^Re-nanocolloid resin for application. Due to the short half-life of ^188^Re (16.9 h), on-site availability is necessary. These challenges limit the treatment primarily to specialized tertiary centers with nuclear medicine capabilities, restricting rural or remote access. In addition, regions without reactor facilities or strict regulatory frameworks may encounter barriers to adoption.

Although the results are encouraging, several limitations reduce the overall strength of the evidence. Most of the included studies were preliminary and lacked control groups, which increased the RoB by introducing selection bias and potentially overestimating treatment efficacy. While sensitivity analyses reduced heterogeneity, residual variability may persist due to differences in follow-up duration, lesion type, lesion depth, prior treatments, clinical settings, study design, target dose, outcome assessment methods, or application protocols. The absence of randomized controlled trials further prevents robust comparisons with established interventions such as Mohs surgery or cryotherapy.

Future studies with standardized reporting are needed to enable formal meta-regression analyses to quantitatively assess the impact of moderators, such as histology, delivered dose, and lesion depth, on treatment outcomes. Future research should prioritize rigorously designed clinical trials with active comparators to validate these findings across broader patient populations. Long-term follow-up is crucial for assessing recurrence rates and cosmetic outcomes beyond 12 months. Incorporating histopathological confirmation before and after treatment would improve diagnostic accuracy and allow for subgroup analyses according to tumor subtype, thereby controlling for potential confounders such as lesion site, covered margin, delivered dose, treatment schedule, and response criteria. Investigation of biomarkers linked to immune activation may also clarify underlying mechanisms and support individualized dosing strategies. Furthermore, evaluating the effectiveness of rhenium-aided skin cancer therapy in both treatment-naïve lesions and those refractory to prior therapies will be important for defining its clinical role.

## 6 Conclusion

Rhenium-aided skin cancer therapy emerges as a potentially valuable non-invasive option for the treatment of skin cancer lesions, offering encouraging response rates and manageable toxicity, even in anatomically complex sites where surgery is limited. Nonetheless, the evidence to date remains preliminary and uncontrolled. Definitive confirmation from well-designed comparative trials is required before this modality can be established as a standard therapeutic option.

## Data Availability

The original contributions presented in the study are included in the article/Supplementary material, further inquiries can be directed to the corresponding author.
