# Supplementary material for: Targeted β-therapy with rhenium-aided therapy for cutaneous lesions: a systematic review and meta-analysis
Source: Front Med (Lausanne). 2025 Nov 27;12:1707729. doi: 10.3389/fmed.2025.1707729 (PMC12695840; doi:10.3389/fmed.2025.1707729)
Supplement: Supplementary file 1 [file Data_Sheet_1.docx]

***Supplementary Table 1. A summary of Reported Adverse Events of Rhenium-Aided Skin Lesion Therapy***

| Study | Side effects |
| --- | --- |
| Cipriani et al. (1) | No complications or other post-interventional problems reported. |
| Cardaci et al. (2) | Overall, 142 events in 32.6% of patients (primarily mild reactions), grade 1 hypopigmentation 60.4%, 6 severe (dermatitis, ulceration, induration, radiation skin injury), 11 events of grade 3-4 (mostly at D 14). No toxicities greater than grade 2 at 12-month. |
| Tietze et al. (3) | Radiodermatitis (97.5%; grade 1, 32.5%, grade 2; 65%), hypopigmentation (49%; 0% at day 14, 25% at months 4, 49% at months 12), pain (12.5%), itching (20%), burning sensation (7.5%). 62.5% of the patients did not report any adverse events at any time point. |
| Zagni et al. (4) | NS |
| Klein et al. (5) | Transient HAMA (9/20 of patients); no dose-limiting toxicities. |
| Chessa et al. (6) | Arborizing-like vessels (in 9/82 patients, due to scarring or relapse) |
| Mokoala et al. (7) | Hypopigmentation (delayed and common). Initial phase (2 weeks post therapy) of erythema and ulceration followed by crusting and scab formation (all patients). |
| Carrozzo e al. (8) | Reported as completely painless, and without any discomfort or collateral effect from the therapy. |
| Bhusari et al. (9) | Radiation dermatitis (1 case due to patch shift); one patient, keloid lesions progress to erythema, ulceration followed by a vitiligo appearance. |
| Carrozzo et al. (10) | Burning sensation, superficial erosions, crusts (early, resolved in 2–3 weeks), managed with topical emollient cream, camomile compress and anti-septic washes. |
| NS: Not Specified, HAMA: Human Anti-Mouse Antibody. | |

***Supplementary Figure 1. Risk-of-bias of the included studies using the ROBINS-I checklist.***

**References**

1. Cipriani C, Desantis M, Dahlhoff G, Brown III SD, Wendler T, Olmeda M, et al. Personalized irradiation therapy for NMSC by rhenium-188 skin cancer therapy: a long-term retrospective study. Journal of Dermatological Treatment. 2022;33(1):969-75.

2. Cardaci G, Baxi S, Vohra S, Allison C, Hong A, Mulholland N, et al. Efficacy, Safety, and Patient Reported Outcomes of Rhenium-Skin Cancer Therapy for Non-Melanoma Skin Cancer: 1-Year Results from the EPIC-Skin Study. Advances in Radiation Oncology. 2025;10(7):101802.

3. Tietze JK, Heuschkel M, Krönert MI, Kurth J, Bandow G, Ojak G, et al. Topical 188Re ionizing radiation therapy exerts high efficacy in curing nonmelanoma skin cancer. Clinical nuclear medicine. 2023;48(10):869-76.

4. Zagni F, Vichi S, Paolani G, Santoro M, Della Gala G, Strolin S, et al. A novel tool for predicting the dose distribution of non‐sealed 188Re (Rhenium) resin in non‐melanoma skin cancers (NMSC) patients. Medical Physics. 2023;50(7):4600-12.

5. Klein M, Lotem M, Peretz T, Zwas ST, Mizrachi S, Liberman Y, et al. Safety and Efficacy of 188-Rhenium-Labeled Antibody to Melanin in Patients with Metastatic Melanoma. Journal of Skin Cancer. 2013;2013(1):828329.

6. Chessa MA, Baraldi C, Savoia F, Maltoni L, Clarizio G, Filippi F, et al. Unsealed188 Rhenium Resin Brachytherapy in Non-Surgical Candidates With Refractory Basal Cell Carcinoma: Clinical Outcomes. Dermatology Practical & Conceptual. 2025;15(1):4993.

7. Mokoala KM, Nonjola L, Moeng T, Corbett C, Magwaza M, Dahlhoff G, et al. Alternative treatment for recurrent keloids: initial clinical experience with Rhenium-188 using a specialized device. European Journal of Nuclear Medicine and Molecular Imaging. 2025:1-8.

8. Carrozzo AM, Sedda AF, Muscardin L, Donati P, Cipriani C. Dermo beta brachytherapy with 188-Re in squamous cell carcinoma of the penis: a new therapy. European journal of dermatology. 2013;23(1):183-8.

9. Bhusari P, Shukla J, Kumar M, Vatsa R, Chhabra A, Palarwar K, et al. Noninvasive treatment of keloid using a customized Re‐188 skin patch. Dermatologic therapy. 2017;30(5):e12515.

10. Carrozzo A, Cipriani C, Donati P, Muscardin L, Sedda A. Dermo Beta Brachytherapy with 188Re in extramammary Paget's disease. Giornale Italiano Di Dermatologia E Venereologia: Organo Ufficiale, Societa Italiana Di Dermatologia E Sifilografia. 2014;149(1):115-21.
